# Opioid-Free Anesthesia and Postoperative Outcomes in Cancer Surgery: A Systematic Review

**DOI:** 10.3390/cancers15010064

**Published:** 2022-12-22

**Authors:** Dario Bugada, Megan Drotar, Simone Finazzi, Giovanni Real, Luca F. Lorini, Patrice Forget

**Affiliations:** 1 Emergency and Intensive Care Department, ASST Papa Giovanni XXIII, 24127 Bergamo, Italy; 2 School of Medicine, Medical Sciences and Nutrition, University of Aberdeen, Aberdeen AB25 2ZD, UK; 3 Department of Health Sciences, University of Milan, 20122 Milan, Italy; 4 Epidemiology Group, Department of Anaesthesia, NHS Grampian, Institute of Applied Health Sciences, School of Medicine, Medical Sciences and Nutrition, University of Aberdeen, Aberdeen AB25 2ZD, UK

**Keywords:** opioid free anesthesia, opioid-based anesthesia, cancer, surgery, postoperative pain, perioperative outcomes, complications, oncologic surgery, cancer patients

## Abstract

**Simple Summary:**

As a significant knowledge gap exists on the effect of opioid-free anesthesia on outcomes of cancer patients receiving surgery, we conducted a systematic review to assess any differences between opioid-free and opioid-based anesthesia. Few studies were included, as the main body of the existing literature in the specific population of oncologic surgery does not reflect high quality standards and displays strong heterogeneity in methodology. Despite encouraging suggestions from the few studies regarding the benefits of opioid-free approaches on the immediate perioperative outcome, more trials are required to accept or reject the superiority of opioid-free anesthesia in patients receiving surgery for cancers.

**Abstract:**

Background: Surgery is an essential component of the treatment of solid tumors, but the perioperative course can be complicated by different factors (including anesthesia). Opioid-free anesthesia (OFA) may mitigate adverse outcomes of opioid-based anesthesia (OBA), but major questions remain on the actual impact in terms of analgesia and the improvement of surgical outcomes. To address this issue, we present a systematic review to evaluate the efficacy of OFA compared to OBA in the specific subset of cancer patients undergoing surgery. Methods: following the Preferred Reporting Items for Systematic reviews and Meta-Analyses (PRISMA), we searched MEDLINE, Embase and the Cochrane CENTRAL Library to include randomized controlled trials (RCTs) on adults undergoing oncological surgery, comparing OFA and OBA up to March 2022. Additional papers were added from the reference lists of identified sources. Papers were manually reviewed by two independent authors to ascertain eligibility and subsequent inclusion in qualitative analysis. Results: only two studies were eligible according to inclusion criteria. It was not possible to perform any meta-analysis. The two studies included patients undergoing prostate and gynecologic surgery on 177 patients, with significant heterogeneity in the outcomes. Conclusions: randomized controlled trial specifically addressed to cancer patients are lacking. A knowledge gap exists, neither confirming nor rejecting the capacity of OFA to improve early postoperative outcomes in cancer surgery. Long-term consequences on specific oncological outcomes are far from being elucidated. We expect a growing body of literature in the coming years. Further studies are required with homogeneous methodology and endpoints.

## 1. Introduction

Surgery is an essential component of the treatment of solid tumors and is often acknowledged as a first line of treatment for cancers. However, the perioperative course can be complicated by pre-existing factors related to the pathology, or by treatments associated with surgery (including anesthesia). Postoperative complications can have a long-term impact on patient outcomes. What may pose a challenge is any adjuvant therapies patients may undergo pre- and post-operatively. For example, immunotherapy, radiotherapy or chemotherapy [1,2]. Anesthesia is an additional factor; some anesthetics interact with the cellular immune system, which poses a challenge to cancer biology and long term outcomes [2]. The evidence on humans remains unclear but, to date, data suggest that in certain cancers, the choice of anesthetic may impact cancer recurrence rate [3]. Furthermore, anesthetic techniques are well known to influence several aspects of perioperative outcomes, including subjective patients’ distress, as well as major morbidity and mortality. Recently, growing interest has been reported for anesthetic techniques that do not rely on opioids, which may be of particular interest in relation to patients receiving oncologic surgery [4,5,6].

Synthetic intravenous opioids were developed in the 1960s and have been used prolifically in general anesthesia. With the advance of knowledge in underlying physiology, the concept of balanced, multimodal anesthesia has developed over the years, with the aim to improve postoperative analgesia. Indeed, balanced anesthesia typically relies on opioids [7]. However, evidence shows that opioids are far from being ideal analgesics, especially when used alone. “Pain” (i.e., an unpleasant sensory and emotional experience, which implies a cognitive perception) is far more likely to happen during general anesthesia. What we call “analgesia” is actually “anti-nociception” [8], a complex phenomenon that should not be obtained by interfering with opioid signaling alone [9]; opioids mainly act on spontaneous pain driven by un-myelinated c-fibers (with no action on “dynamic” pain that comes from A-delta fibers). Opioids only partially account for nociception and should not, therefore, be considered as a comprehensive approach to surgical anesthesia.

On the other hand, opioids are indisputably associated with undesirable side effects, such as respiratory depression, postoperative ileus and postoperative nausea and vomiting (PONV), though consequently improving overall patient recovery. Recent evidence has shown that opioids are capable of interfering with immunological processes [10] and contribute to the paradoxical creation of postoperative hyperalgesia and tolerance [11,12,13]. 

The concept of OFA may be summarized as “multimodal anesthesia” to supply for OBA [4]. A rational strategy implies: (1) combining anti-nociceptive agents to target different circuits involved in nociceptive transmission and to provide pain control and reduce postoperative hyperalgesia, while explicitly exploiting the secondary effects of anti-nociceptive agents to reduce the doses of hypnotics, maintain hemodynamic stability and improve perioperative outcomes. These strategies are of prominent interest in relation to the evolution of the concept of Enhanced Recovery After Surgery (ERAS), in which several items are proposed to reduce side effect from opioids, improve patient’s reported outcomes (PROMs) and promote effective rehabilitation.

However, OFA implementation remains difficult because of the lack of consensus. Although everyone agrees that OFA may be safe [14], major questions remain on the actual impact in terms of analgesia and improvement of surgical outcomes [4,6]. 

More specifically, a gap in the literature exists concerning outcomes following opioid free anesthesia during oncological surgery. The current literature is focused broadly on OFA vs. OBA outcomes in mixed populations, owing to the broad range of cancers and associated surgeries being examined, plus there is the consequent disparities in postoperative outcomes and associated treatments which may have an impact on postoperative recovery (including radiotherapy, immunotherapy and neoadjuvant chemotherapy [1].

To address this issue, we sought to conduct a systematic review to evaluate the efficacy of OFA compared to OBA in the specific subset of cancer patients undergoing surgery.

## 2. Materials and Methods

### 2.1. Data Sources and Search Strategy

This systematic review was conducted from January to March 2022, following the Preferred Reporting Items for Systematic reviews and Meta-Analyses (PRISMA) [15]. The protocol consisted in establishing eligibility criteria using the Population, Intervention, Comparison, Outcomes and Study (PICOS) framework and generating an appropriate search strategy. The protocol was registered on PROSPERO on 16 March 2022 (registration number CRD42022316813). 

We searched MEDLINE, Embase and the Cochrane CENTRAL Library with no restrictions on publication language or status, using the following keywords: “opioid free” AND/OR “cancer surgery” AND/OR “cancer surgery” AND/OR “oncology” AND/OR “surgery”, “opioid free adj4 cancer surgery”, “opioid free anesthesia” AND/OR “cancer surgery”, “opioid free adj3 surgery” AND/OR “opioid free adj4 cancer surgery”, “oncological surgery”, “opioid free anesthesia”. Additional papers were taken from the reference lists from papers of interest and manually reviewed. 

### 2.2. Eligibility Criteria

For a paper to be considered eligible for this scoping review, a set of inclusion and exclusion criteria were generated through the PICOS framework:

Patient: adults (18–65 years) undergoing oncological surgery;

Intervention: opioid-free anesthesia;

Comparison: opioid-based anesthesia;

Outcome(s): Primary: incidence of postoperative nausea and vomiting (PONV), analgesic consumption after 24 h, quality of recovery (QoR), pain profiles and return to intended oncologic treatment (RIOT). Additional: adverse events and overall survival;

#### Study: Randomized Controlled Trials (RCTs)

This stipulated that the only studies to be considered had to be randomized controlled trials (RCTs), the participants in each study were adult patients undergoing elective cancer surgery under opioid-free anesthesia, specific postoperative outcomes relating to pain, adverse events and overall survival/recurrence rate were measured, and these same outcomes were measured against cancer patients who had undergone the same surgery but under opioid anesthesia.

The initial articles found were screened to see if they met the eligibility criteria before undergoing a further screening process by two independent parties (MGD and PF), in order to ensure that eligibility criteria were being met without selection bias. The PICOS framework was used as a guide to discern trial eligibility. Information about the trials (first author, year of publication, country, number of groups in the study and sponsorship), participants (characteristics of the population and number of patients randomized and analyzed) and experimental intervention (drug used and doses) were extracted. Two independent reviewers (S.F. and G.R.) assessed the quality of the trial methodology with the Cochrane Risk of Bias tool, and any discrepancies were resolved by consensus.

## 3. Results

### 3.1. Search Results

**Figure 1:**
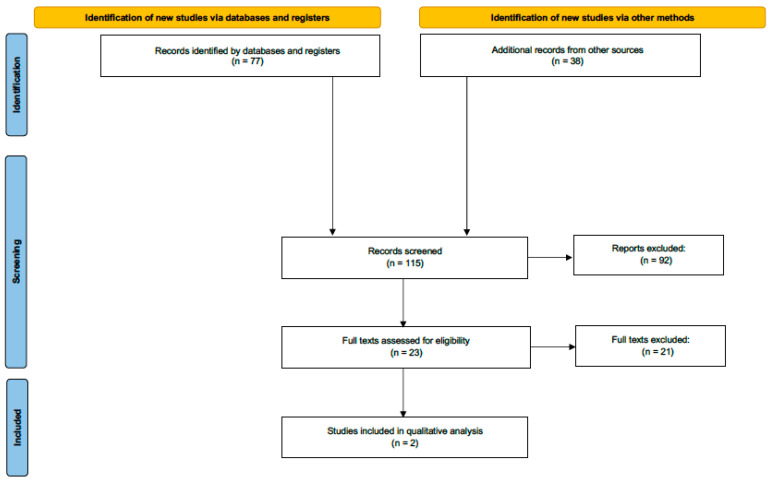
PRISMA flow diagram summarizing the retrieved, included and the excluded studies. PRISMA, Preferred Reporting Items for Systematic Reviews and Meta-Analyses.

From the relevant sources identified from database searches for further screening (n = 77), the reference lists were examined manually to find any additional relevant literature. From this process, 38 additional papers were identified and included in the screening process. Finally, 115 papers were suitable to undergo further screening. The PICOS framework was used to identify eligible papers and exclude those which were unsuitable—92 studies were removed on this basis. From here, the remaining 23 RCTs were assessed by two reviewers (MGD and PF) for eligibility and inclusion. Disparities in reviewer exclusion were resolved through discussion. Of these papers, 21 were excluded which left two trials suitable for qualitative synthesis (Figure 1). 

Two studies excluded from the final analysis during full-text review are ongoing or not yet published. 

### 3.2. Outcomes

Hontoir et al. investigated, in a study carried out in Belgium, the effect of a peri-operative opioid-free approach on postoperative patient comfort in patients undergoing breast cancer surgery. The primary outcome of this study was the postoperative patient comfort, assessed by the Quality-of-Recovery—QoR-40—questionnaire. As a secondary outcome, pain intensity was evaluated using a numeric rating scale score (NRS), as well as postoperative piritramide consumption. The OFA approach resulted superiorly when compared to standard OBA approach for all outcomes; a statistically significant difference was retrieved for QoR-40 scoring (especially in the emotional support and the pain component) in pain intensity at 24 h after surgery and additional opioid consumption (8.1 mg—SD 6.6 in the OFA group, and 13.1 mg—SD 9.4 in the OBA group). 

Rangel et al. evaluated, in a study conducted in Brazil, whether opioids used during prostatectomy can affect biochemical recurrence-free survival. Among the secondary outcomes of the study, postoperative pain (by NRS scale) in the PACU and the need for additional opioid requirements (PCA with morphine) in PACU were also evaluated. No differences in pain and morphine consumption were retrieved, as well as no differences in patients’ satisfaction, between OFA and OBA.

In both studies, there was no difference in AEs. In the study by Hontoir et al. improved sedation measured by the Ramsay Scale was reported at 60 min in the PACU. No differences in the final LOS were reported by either study.

Given the small number of studies and the differences between them in terms of the reported outcomes, it was impossible to conduct a meta-analysis (Table 1).

### 3.3. Risk of Bias Assessment

**Figure 2:**
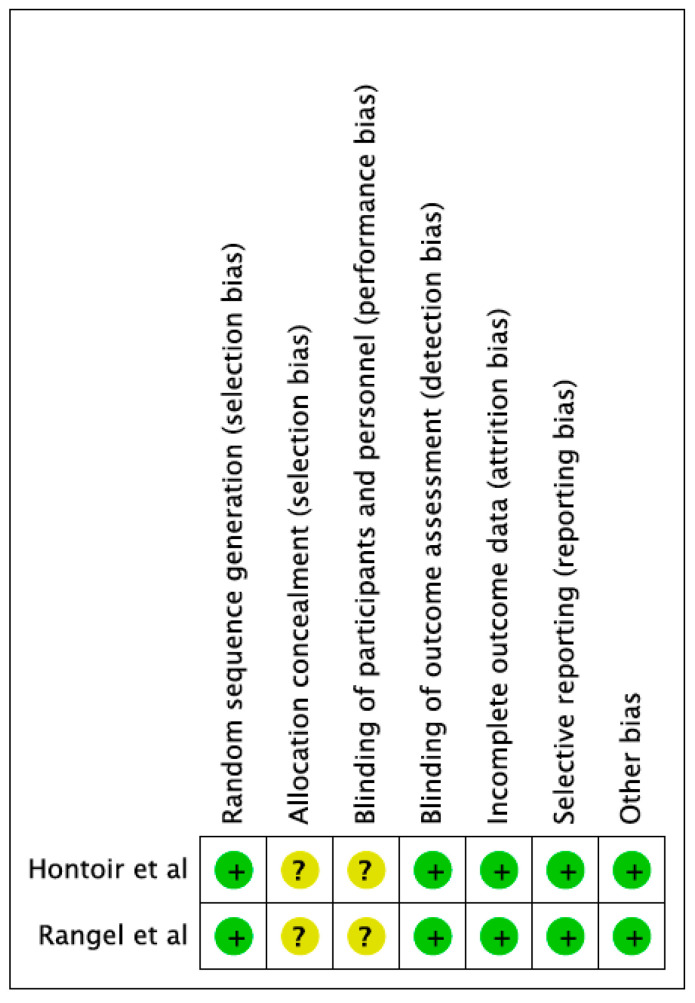
Risk of bias summary. Review authors’ judgements about each risk of bias item for each included study [16,17].

All studies were considered of high quality for all possible bias, except for allocation and blinding (moderate risk) that are not clearly and exhaustively described (Figure 2).

## 4. Discussion

The most relevant finding from this systematic review was the lack of suitably robust randomized controlled trials measuring relevant postoperative outcomes in cancer patients. Although there is a notable interest in OFA, with over 4000 registered trials in 2019 [18], and a rich selection of the literature in the form of review articles and retrospective studies, there remains a deficit in trials on both OFA and cancer surgery. The reason why our tentative metanalysis was not possible is primarily the lack of relevant studies to be included. 

Also, the few eligible randomized controlled trials identified were homogeneous regarding the types of cancer and patients involved. Notably, much of the literature surrounding OFA in cancer surgery focuses on very few types of cancer, breast and prostate malignancies being the most commonly studied. This is not coincidental, as they are the most common sex-linked cancers for women and men, respectively [19,20]. While they are suitable model, being the most common cancers encountered, they do not account for any differences which may be found in other types of cancer, particularly those of an endocrine origin, where there is potential for anesthetic agents to interact with the cancer biology in a different way [3]. From the existing literature, there is a high degree of variation in terms of how specific patient populations react to opioids or other anesthetic treatments under certain conditions. What is not addressed is whether certain cancers affect outcomes and if OFA mitigates against adverse postoperative outcomes in these scenarios. Alongside cancers examined, a lack of variation in surgical procedures is limiting in the sense that the measured postoperative outcomes are relatively homogenous—either because OFA produces uniform outcomes or because it is best suited to certain types of surgery. 

However, some outcomes are of major importance in all kinds of surgeries and have been considered for years as the main indicators of the quality of perioperative management. The primary reason for the interest in OFA its efficacy in providing better analgesia with fewer side effects (namely opioid-related side effects). Previous RCTs and metanalysis in the general population suggest that OFA is promising in this sense [21,22]. Recent studies have often shown that patients treated with OFA had less postoperative nausea and vomiting (PONV) in the postoperative period [21,22], despite no differences being observed in terms of pain and opioid use. PONV has always been regarded as a major outcome in the context of enhanced recovery after surgery (ERAS), and is included in the list of the main patient reported outcomes (PROs) that define the subjective experience of the perioperative period by the patient [4]. In the context of cancer patients (where PONV may already be an issue before surgery because of concomitant treatment) anesthetic strategies that reduce opioid consumption and PONV are of greater interest. Unfortunately, it is impossible at this time to determine this reduction in the specific setting of oncologic patients. In the two studies available for inclusion in this systematic review, none reported PONV as an outcome [16,17].

According to the studies included in our review, potential benefits given by OFA in terms of analgesia still need to be clarified (at least in the context of cancer surgery). In one study (breast cancer) analgesia was superior (with less opioid consumption) in the OFA group [16], while in the other one (prostate cancer) no difference existed [17] in morphine consumption (despite more patients having moderate–severe pain in PACU with OFA). As few data are available, and the fact that they only came as secondary outcomes from both studies, the need for further study is highlighted by our research. Since pain is a major item for the definition of quality in perioperative care, new studies should be focused on pain and opioid consumption after surgery. Furthermore, we stress the importance for new research to focus on specific type of cancers (and surgery) since they may inherently be associated with different degrees of pain. Although OFA may be an effective intervention in most cases, it might not be an entirely suitable one depending on the type of cancer. There is insufficient supporting evidence at the moment to indicate whether this is the case. By measuring similar outcomes in different types of cancer—and, by extension, types of surgery—the mechanisms of OFA can be placed into a broader context, and it may be determined whether it is a suitable intervention to be implemented in oncological surgery. Depending on the type of cancer and the outcome of interest (immediate recovery, patients’ comfort endpoints or long-term outcomes), OFA may be promising for pain management, but also a reasonable approach for specific aims relevant for the oncologic patient [5].

Nevertheless, OFA (more than OBA) aims to reduce stress response after surgery. Anesthesia alone is insufficient to modulate the stress response [23]; analgesia also contributes to this mediation. Use of central nervous system (CNS) agonists, traditionally opioids such as fentanyl and remifentanil, are effective at inhibiting the systemic nociceptive pathways which are responsible for triggering the immune response to surgery [24]. By doing this, the surgical stress response is not eliminated but reduced significantly. This reduced stress response is important, as a state of catabolism left unchecked can lead to issues including infection, starvation, hypothermia, thrombosis, hemorrhage, hyperalgesia and inflammation, both intra- and post-operatively [25]. In cases of oncological surgery, it is particularly important that immunological stressors are mitigated against, not only in the interests of patient safety, postoperative care and recovery, but also to ensure that surgical stress does not exacerbate cancer biology and contribute to micro-metastases, which has been observed in certain types of endocrine cancers [26]. The use of alpha-2 agonists, lidocaine, regional anesthesia and ketamine in OFA regimens are common (despite the need for standardization) and act together, providing reduced stress response and increasing inflammatory–immune homeostasis. They may also add significant advantages over common opioid-based approaches [27,28,29,30]. The trials examined over the course of this systematic review did not take surgical stress into account alongside the OFA versus OBA anesthetic regimens in cancer surgery. However, it would be interesting to measure if administration of OFA is as effective as or more effective than the traditional opioid route in mediating surgical stress, alongside maintenance of anesthesia, and whether this improves postoperative cancer outcomes as a result.

Notably, after the full text was reviewed for final selection, we retrieved two studies that are still ongoing or not published. According to our available (CTRI/2018/04/013335—India Clinical Trial Registry; ClinicalTrial.gov NCT04390698) these two studies would satisfy inclusion criteria relevant for comparison between OBA and OFA. Once data from these studies are accessible, additional information will probably be available for further literature search and metanalysis.

## 5. Conclusions

Despite the great interest in OFA, relevant randomized controlled trials specifically addressed to cancer patients are lacking. To date, a knowledge gap exists, neither confirming nor rejecting the capacity of OFA to improve outcomes in cancer surgery. Long-term consequences on specific oncological outcomes are far from being elucidated. However, we expect a growing body of literature in the next few years.

## Figures and Tables

**Table 1:** Characteristics of the two studies included after systematic literature search. OFA: Opioid-Free Anesthesia; PACU: Post-Anesthetic Care Unit; AEs: Adverse Events; LOS: Length of Stay.

Author	Patient	Intervention	Comparator	Outcome(s)	Results
Hontoir et al., 2016 [16]	Adult female, breast cancer surgery (total analysed n = 34)	Opioid-free anaesthesia (n = 31): ketamine, lidocaine, clonidine.	Opioid general anaesthesia (n = 33)	Postoperative patient comfort and analgesic, pain and consumption (24 h)	OFA provides better patient satisfaction * and analgesia in PACU and at 24 h. * Statistical but not clinical relevance according to the authors primary hypothesis
Rangel et al., 2021 [17]	Adult male, prostate cancer surgery (total analysed n = 143)	Opioid-free anaesthesia (n = 72): propofol, ketamine, lidocaine, cisatracurium and TAP block with ropivacaine	Opioid general anaesthesia (n = 71): propofol, ketamine, lidocaine, cisatracurium, TAP block with saline and fentanyl	biochemical recurrence. Postoperative pain, analgesic requirement, time to discharge; survival measured over 6 months to 2 years.	No differences in pain and morphine consumption in PACU, despite higher incidence of moderate/severe pain in the OFA group. No differences in patients’ satisfaction. No difference in AEs, PACU discharge and LOS
